# Creating a college adjustment index score for student veterans with and without disabilities

**DOI:** 10.3389/fpsyt.2022.1020232

**Published:** 2022-10-26

**Authors:** Emre Umucu

**Affiliations:** Department of Counseling, Educational Psychology, and Special Education, Michigan State University, East Lansing, MI, United States

**Keywords:** positive psychiatry, positive psychology, college adjustment, veterans, self-efficacy, Tinto’s model

## Abstract

Higher education is a critical public health tool to achieve economic success, upward mobility, and improved quality of life. Yet, certain groups of students, including student veterans with and without disabilities (SVDs), are at high risk for course failure and dropout, partially due to challenges related to college adjustment. The purpose of this study is to develop a new college adjustment index score for SVDs. We had a total of 4 different research studies to examine the psychometric properties of our college adjustment index score. After conducting a series of psychometric analyses, we selected a total of 18-items. This 18-item tool may help clinicians and researchers conceptualize college adjustment among students through the lens of integrative Tinto’s model and positive psychology approaches. Our psychometric analyses revealed that this index tool is brief, reliable, and valid tool to capture college adjustment in SVDs.

## Introduction

Higher education is a critical public health tool to achieve economic success, upward mobility, and improved quality of life (1–3). This stance aligns well with research documenting that education increases average lifetime earnings, societal contributions, healthier lifestyle, and reduces poverty, tendency to commit crimes, and unemployment (4). Yet, certain groups of students, including student veterans with and without disabilities (SVDs), are at high risk for course failure and dropout, and this problem can be attributed to transitioning difficulties from military to civic life and then to college, experiencing severe disabilities and chronic conditions associated with military service, and facing with high levels of academic, social, financial, and psychosocial stressors (5–10).

### Challenges during higher education for student veterans

Although the post–9/11 GI Bill helps many Veterans with disabilities attend school, more than 50% of SVDs are first-generation college students with minimal academic preparation compared to traditional civilian students so that SVDs experience additional challenges in coping with higher education demands (11). Many SVDs also experience academic-, transition-, and disability-related challenges and stressors, which is a significant rehabilitation, education, and public health concern (12). Literature has well-documented that factors contributing to college life and adjustment can be complex and cannot be reduced to a single factor, such as academic problems (8). For example, one research reported that college adjustment can be conceptualized as the absence of common problems (i.e., academic problems, psychological and physical health problems, substance use problems, and interpersonal relationship problems) (13). Similarly, Baker et al. (14) reported that college life adjustment consists of four subfactors including academic adjustment, social adjustment, personal-emotional adjustment, and goal commitment/institutional adjustment. The consensus is that college adjustment is a multidimensional concept with multiple components.

Like any traditional student, SVDs also experience substantial challenges related to their college adjustment [e.g., (15, 16)]. However, the magnitude of negative effects of these challenges could be stronger due to unique military-related experiences. For example, due to their previous military service, SVDs may start college later in their life, creating a significant age difference between civilian students and SVDs, which ultimately increase psychosocial and academic challenges among SVDs (5, 17, 18). Literature revealed that student veterans are more likely to be older than the 18–22 years of traditional college-age (19). In addition to age differences, unstructured college environment may be difficult to adjust for SVDs after spending time in a structured military environment. Finally, SVDs could be a transfer or part-time student or could work part-time or full-time, which all can affect their college adjustment (19, 20).

In addition to challenges and stressors reported above, disability/ies and chronic conditions associated with deployment may further negatively affect college life in SVDs (5, 12). Previous research has well-documented that physical, cognitive, and psychiatric disabilities are very common in student veterans (5, 8, 16, 17, 21–25). One research revealed that 46% of student Veteran reported having PTSD-related symptoms (21). Thomas et al. (26) reported that a significant amount of student veterans (44%) reported an existing diagnosis. Suicidal ideation is also common among student Veterans, with up to 46% of student service members/Veterans reported lifetime incidence of suicidal ideation (15, 21, 27). Besides, research revealed that 35% of student veterans experienced severe anxiety and 24% experienced severe depression (21). The 2020 Student Veterans of America Census Survey revealed that 64.5% of student veterans reported having a VA disability rating and about 4% were in the process of submitting a disability claim (22). Same survey also revealed that more than 50% of these SVDs had a 70% or more VA disability rating (22). Besides, about 75% of these disabled student veterans reported that their school was impacted by their disability (22).

Overall, literature revealed that disability and disability-related symptoms significantly increase academic and career related concerns (e.g., course dropout), substance use problems (e.g., alcohol abuse), relationship problems (e.g., social isolation, loneliness), physical health concerns (e.g., insomnia), and psychological health problems (e.g., depression) in student veterans (5, 21, 22), which may cause dropout and/or late or no academic degree, and eventually poor employment and wellbeing outcomes.

### Emotional immunity among student veterans in higher education

The majority of literature on higher education success focuses on academic aspects of higher education and underestimate emotional aspects of college life, which is problematic given emotional health, or we call “emotional immunity” is a critical aspect of college and academic success (28, 29). In a meta-analysis study, researchers found that psychosocial health scores (e.g., stress) were correlated with GPA (30). One research, interestingly, reported that “depressive symptoms,” “exposure to stressful life,” and “antisocial behaviors” were three consistent predictors of student retention (31). Besides, Robbins et al. (31) also highlighted the importance of protective factors (e.g., social support) in college retention. Researchers also found that academic achievement was negatively correlated with depression and anxiety and positively correlated with gratitude, social connectedness, and life satisfaction (32). Renshaw et al. (32) also reported that emotional wellbeing is a strong predictor of college student outcomes.

Since emotional injuries (e.g., PTSD) are as common as physical injuries in student veterans (5, 16, 18, 19), the majority of research has focused on psychopathology and symptomology in SVDs (8). Due to any symptom (e.g., negative emotions) associated with a service-connected disability and chronic condition (e.g., TBI), SVDs may have difficulties in completing their academic assignments, participating in school activities, and building positive relationships [e.g., (5, 16)]. Emotional immunity or strengths have not been examined thoroughly in SVDs, which is problematic to identify protective emotional factors in this sample.

Recent research examined emotions and wellbeing in SVDs disabilities and reported that higher levels of emotional character strengths were associated with higher levels GPA, optimism, hope, and resilience and lower levels of loneliness, depression, anxiety, and stress in this population (8). Researchers also examined whether pillars of wellbeing mediate the relationship between PTSD and college life adjustment and reported that positive emotions and accomplishment mediated the relationship between PTSD and college life in SVDs (12). One research examined flourishing as an emotional concept and reported that those with higher levels of flourishing had higher levels of resilience, life satisfaction, and wellbeing and lower levels of stress, anxiety, and depression in SVDs (33). Previous research, overall, revealed that emotional strengths and immunity should be carefully screened and measured in this body of students to further help them achieve their psychosocial and education goals.

### Help-seeking behaviors among veterans in higher education

Student veterans with and without disabilities experience high levels of psychosocial and academic stressors and challenges; however, SVDs are not aware of their disabilities; are not seeking help; or do not have access to seek help (9, 34). SVDs may perceive help-seeking as a weakness, a challenge for their self-esteem, and a sense of inadequacy, thus increasing self-stigma to seeking mental health services (35). In addition, due to negative beliefs about mental health and treatments, SVDs may have high levels of treatment non-compliance and non-adherence (8, 36, 37). Due to lower levels of help-seeking behaviors, untreated disability symptoms in addition to academic stressors often compromise stability, prevent SVDs from successfully graduating from college and ultimately limits the their success (8, 33, 38–40). This is very concerning given lower levels of educational attainment is closely associated with increased rates of unemployment and poverty, creating significant health disparities and challenges for SVDs (41).

Student veterans with and without disabilities may also endorse negative beliefs about treatment approaches (36) given university counseling services heavily focus on treating psychopathology (e.g., depression symptoms), often overlooking the value of strength-based counseling (e.g., increasing positive emotions). This may reinforces the negative stereotypes about interventions given veteran culture highly endorses core values of toughness and strengths (42). Therefore, it is important to measure and understand whether SVDs are willing to seek help from professionals when they face with psychosocial and academic stressors.

### Existing college adjustment scales

College adjustment has been a major interest among education and psychology researchers. There is a consensus that college adjustment is multidimensional construct and consists of multiple subconstructs [e.g., (13, 14)]. Although this concept has been a major interest among researchers, there are only few scales measuring this construct. First, the *Student Adaptation to College Questionnaire* (SACQ) (14, 43, 44), one of the most widely used questionnaires, was developed to measure the college adaptation among students. The SACQ consisted of 67-items measuring four subscales named academic adjustment, social adjustment, personal-emotional adjustment, and goal commitment/institutional adjustment. SACQ is not brief and not very accessible due to associated fee. Besides, some researcher reported that “without explicating the theory behind (SACQ)’s development and without clearly defining adjustment other than how well students meet the various role demands, their (44) initial validation efforts provided evidence of criterion related validity” (p.93) (45). Although its certain weaknesses, the SACQ has still been one of the most widely used tool of college adjustment.

Hoffman and Weiss (13) developed an inventory, called the *Inventory of Common Problems* (ICP), to measure and conceptualize college students’ problems. This scale consists of a total of 24-items measuring six categories of common problems (i.e., depression, anxiety, substance use problems, interpersonal relationship problems, physical health problems, and academic problems) (13). Based on authors’ initial findings, one can claim that the ICP is psychometrically sound and relatively shorter scale. However, authors reported that substance use problems subscale was found to have low reliability and validity compared to other five subscales. Although this scale is shorter than the SACQ, not a clear theoretical orientation was applied when developing the ICP. In addition, like SACQ, this scale also does not cover any help-seeking behaviors among college students which is critical to provide best services to college students.

### A college adjustment index initiative for student veterans

This study does not aim to create a new scale or index score from scratch, instead we focused on available and widely used tools to create a college adjustment index score. First, we conceptualize *college adjustment* as an achievement of the final state of highest student–college congruence. The existing scales to measure college adjustment in the education and psychology literature present several limitations. For example, the *SACQ* captures four domains of the college adjustment, but does not directly assess potential problems experienced by the students. Besides, the SACQ has been widely used only in North American students, limiting its generalizability to other cultures (46). Besides, the SACQ 67-items which is comparatively lengthy if administering the scale in a time-limited clinical and research setting. Furthermore, both the *SACQ* and *ICP* did not have a clear theoretical background or orientation on student retention or adjustment while developing items. Given student retention is key in higher education (47), and college adjustment is predictor of retention, we aimed to develop a new, brief, theoretically oriented, and psychometrically sound index score to measure college adjustment in students, including SVDs.

Based on Tinto’s integration framework (47–49), students will not dropout if they are connected and committed to the academic and social life of the institution (50). Tinto (49) reported that although some students are successful to cope with problems of adjusting to the social and intellectual life of the higher education, many find this adjustment measurably more challenging. This adjustment approach is relatively similar to resilience and strengths approach in positive psychology. According to Tinto (51), psychosocial factors play a critical role during college adjustment since they help students integrate in academic and social environment. In their work, Napoli and Wortman (52) extended and further refined Tinto’s model by examining the mediational influences of a comprehensive set of psychosocial measures (e.g., wellbeing) on the constructs within Tinto’s model. Their results revealed that psychosocial measures had both direct and indirect effects on college persistence (52). Although Tinto’s model has widely been applied in education research, no research, to our knowledge, has been examined Tinto’s model for SVDs.

Recently, researchers have also examined whether positive psychology factors are predictors of student retention and academic success in higher education. One research examined resilience, academic self-concept, and college adjustment in college students and reported that resilience and academic self-concept were both significant predictors of college adjustment for college students (53). Another work examined hope and college adjustment and reported that hope was positively related to college adjustment in college students (54). A recent work examined wellbeing, PTSD, and college adjustment in SVDs and reported that positive emotions and sense of accomplishments mediated the relationship between PTSD and college adjustment (12). A similar research (8) reported that emotional strengths were positively associated with GPA in SVDs. Finally, Umucu (39) examined positive psychology model as a college adjustment and wellbeing model for SVD and found that positive psychology is a promising approach for SVDs given it focuses on strengths.

To our knowledge, Tinto’s model and positive psychology has never been used together to examine college adjustment and retention in college students including SVDs. As reported, university counseling services focus on treating psychopathology (e.g., depression symptoms), often overlooking the value of strength-based counseling (e.g., increasing positive emotions). This approach could be problematic given SVDs endorse negative beliefs about treatment approaches, reinforcing the negative stereotypes about interventions given veteran culture highly endorses core values of toughness and strengths (42). Therefore, the purpose of this study is to develop a new college adjustment index score for SVDs.

## Materials and methods

### Study 1

Upon the IRB approval of the Study 1, the researcher reached out to directors of student veteran programs across the USA to recruit participants. SVDs were recruited from several universities across the country. Participants signed the online consent form before they started the survey. Participants were sent a $15 gift card upon completing the survey. A total of 205 SVDs (*M*_*age*_ = 29.32, SD = 8.02) were recruited for the Study 1. The majority of sample was male (71.7%), white (80.5%), followed by 10.2% Hispanic/Latino, 2.9% African American, 2.4% bi-racial, 1.5% Asian, and 2.5% Other. Forty percent of participants served in the Army, followed by the Air Force (22.4%), Marine Corps (20.5%), Navy (16.6%), and Coast Guard (0.5%). Thirty-nine percent of participants had service-connected disabilities.

#### Measures

The Study 1 was used to generate the item pool from other psychometrically sound and widely used scales. These variables and domains are determined based on our previous work. *Positive emotion*, *engagement*, *relationships*, *meaning*, and *accomplishment* were measured by the *PERMA-Profiler* (55). Each pillar of the wellbeing consists of three-items, totaling 15-items. The reported Cronbach’s alpha for the subscale scores ranged from 0.71 to 0.89 for positive emotion, 0.60 to 0.81 for engagement, 0.75 to 0.85 for relationships, 0.85 to 0.92 for meaning, and 0.70 to 0.86 for accomplishment subscale scores (55). *Resilience* was measured using the six-item *Brief Resilience Scale* (BRS) (56). The Cronbach’s alpha for the BRS ranged from 0.80 to 0.91 in previous research (56). *Optimism* was measured using the six-item of the *Life Orientation Task-Revised* (LOT-R) (57). The Cronbach’s alpha of the LOT-R has been reported to be 0.78 (57). *College problems* were measured by the four subscales (i.e., academic problems, interpersonal relationship problems, substance use problems, and physical health problems) of the ICP (13). Each subscale consists of four-items, totaling 16-items. The internal consistency reliability coefficients were found to be 0.71 for academic problems subscale, 0.67 for interpersonal problems subscale, 0.53 for physical health problems subscale, and 0.45 for substance-use subscale (13). *Depression and anxiety* were measured by the four-item *Patient Health Questionnaire for Depression and Anxiety* (PHQ-4) (58). The Cronbach’s alpha coefficient for the scale was reported to be 0.85 (58). *Stigma related to seeking psychological help* was measured with the *Self-Stigma of Seeking Help* (SSOSH) scale (59). The Cronbach’s alpha reliability coefficient was reported to be 0.91 (59). Please see Table 1 for all items and details.

**TABLE 1:** Item pool and initial item characteristics.

Construct	FL	ITC	AID	SE	LS	ST	LNL	DIS	# Criteria
**PERMA (55)**									
PE1. In general, how often do you feel joyful?^+^	**0.896**	**0.829**	**0.856**	0.431*	0.578*	−0.442*	−0.536*	−0.225*	**7^+^**
PE2. In general, how often do you feel positive?	**0.882**	**0.820**	**0.864**	0.496*	0.554*	−0.481*	−0.499*	−0.223*	6
PE3. In general, to what extent do you feel contented?	0.851	0.799	0.883	0.420*	0.624*	−0.526*	−0.551*	−0.186*	3
EN1. How often do you become absorbed in what you are doing?	**0.600**	**0.407**	**0.447**	0.445*	0.252*	−0.290*	−0.236*	−0.131	7
EN2. In general, to what extent do you feel excited and interested in things?^+^	**0.693**	**0.428**	**0.378**	0.462*	0.585*	−0.449*	−0.581*	−0.259*	**8^+^**
EN3. How often do you lose track of time while doing something you enjoy?	0.417	0.322	0.576	0.229*	0.169*	−0.149*	−0.169*	−0.021	0
REL1. To what extent do you receive help and support from others when you need it?	0.667	0.628	0.886	0.332*	0.512*	−0.391*	−0.430*	−0.165*	1
REL2. To what extent do you feel loved?^+^	**0.939**	**0.804**	**0.721**	0.433*	0.564*	−0.483*	−0.505*	−0.135	**7^++^**
REL3. How satisfied are you with your personal relationships?	**0.847**	**0.751**	**0.770**	0.428*	0.624*	−0.542*	−0.563*	−0.127	7
ME1. In general, to what extent do you lead a purposeful and meaningful life?^+^	**0.875**	**0.824**	**0.882**	0.522*	0.586*	−0.456*	−0.458*	−0.218*	**8^+^**
ME2. In general, to what extent do you feel that what you do in your life is valuable and worthwhile?	**0.949**	**0.872**	**0.842**	0.469*	0.510*	−0.435*	−0.449*	−0.205*	6
ME3. To what extent do you generally feel you have a sense of direction in your life?	0.832	0.794	0.906	0.543*	0.492*	−0.475*	−0.429*	−0.162*	2
ACC1. How much of the time do you feel you are making progress toward accomplishing your goals?	**0.785**	**0.684**	**0.712**	0.537*	0.451*	−0.447*	−0.323*	−0.147*	6
ACC2. How often do you achieve the important goals you have set for yourself?^+^	**0.874**	**0.727**	**0.665**	0.613*	0.483*	−0.472*	−0.392*	−0.182*	**8^+^**
ACC3. How often are you able to handle your responsibilities?	0.647	0.585	0.814	0.521*	0.417*	−0.540*	−0.287*	−0.181*	2
**Resilience (56)**									
RES1. I tend to bounce back quickly after hard times.^+^	**0.748**	**0.679**	**0.811**	0.317*	0.449*	−0.438*	−0.286*	−0.111	**7^+^**
RES2. I have a hard time making it through stressful events.	0.631	0.570	0.831	0.113	0.257*	−0.367*	−0.178*	−0.081	0
RES3. It does not take me long to recover from a stressful event.	0.651	0.585	0.827	0.237*	0.350*	−0.361*	−0.205*	−0.191*	2
RES4. It is hard for me to snap back when something bad happens.^+^	**0.755**	**0.684**	**0.808**	0.267*	0.432*	−0.420*	−0.271*	−0.081	**6^+^**
RES5. I usually come through difficult times with little trouble.	0.623	0.567	0.830	0.249*	0.264*	−0.425*	−0.272*	−0.173*	4
RES6. I tend to take a long time to get over set-backs in my life.	**0.752**	**0.681**	**0.808**	0.209*	0.315*	−0.411*	−0.350*	−0.149*	5
**Optimism (57)**									
OPT1. In uncertain times, I usually expect the best.	0.615	0.565	0.851	0.319	0.343*	−0.325*	−0.317*	−0.019	0
OPT2. If something can go wrong for me, it will.	0.718	0.656	0.834	0.262*	0.427*	−0.414*	−0.305*	−0.032	1
OPT3. I’m always optimistic about my future.^+^	**0.726**	**0.669**	**0.832**	0.472*	0.592*	−0.499*	−0.434*	−0.129	7^+^
OPT4. I hardly ever expect things to go my way.	0.709	0.646	0.836	0.204*	0.410*	−0.433*	−0.406*	0.057	1
OPT5. I rarely count on good things happening to me.	**0.747**	**0.681**	**0.830**	0.360*	0.515*	−0.448*	−0.344*	−0.067	6
OPT6. Overall, I expect more good things to happen to me than bad.^+^	**0.748**	**0.680**	**0.830**	0.417*	0.509*	−0.495*	−0.328*	−0.101	**6^++^**
**College problems (13)**									
AP1. Academic problems?	0.631	0.542	0.751	−0.494*	−0.398*	0.428*	0.342*	0.125	2
AP2. Difficulty caring about or concentrating on studies?^+^	**0.744**	**0.645**	**0.698**	−0.416*	−0.422*	0.421*	0.354*	0.214*	**6^+^**
AP3. Indecision or concern about choice of career or major?	0.505	0.450	0.795	−0.273*	−0.277*	0.319*	0.247*	0.123	0
AP4. Feeling like I’m not doing as well at school as I should?^+^	**0.880**	**0.726**	**0.649**	−0.512*	−0.425*	0.491*	0.429*	0.121	**7^+^**
IP1. Problems with romantic or sexual relationships?^+^	**0.709**	**0.611**	**0.724**	−0.304*	−0.410*	0.482*	0.419*	0.200*	**5^+^**
IP2. Family problems?	0.653	0.563	0.747	−0.245*	−0.404*	0.466*	0.363*	0.196*	5
IP3. Difficulty getting along with others?	0.690	0.591	0.735	−0.350*	−0.353*	0.486*	0.501*	0.155*	3
IP4. Feeling lonely or isolated?^+^	**0.719**	**0.611**	**0.724**	−0.362*	−0.485*	0.501*	0.630*	0.223*	**8^+^**
PP1. Physical health problems?^+^	**0.839**	**0.723**	**0.749**	−0.298*	−0.462*	0.458*	0.322*	0.367*	**5^+^**
PP2. Headaches, faintness, or dizziness?	0.676	0.601	0.800	−0.398*	−0.396*	0.420*	0.348*	0.284*	0
PP3. Trouble sleeping?^+^	**0.727**	**0.653**	**0.784**	−0.367*	−0.462*	0.475*	0.389*	0.416*	**7^+^**
PP4. Eating, appetite, or weight problems?	0.722	0.644	0.781	−0.358*	−0.510*	0.470*	0.384*	0.244*	3
SP1. My use of alcohol?^+^	0.310	0.273	0.623	−0.174*	−0.199*	0.265*	0.266*	0.158*	**5^+^**
SP2. My use of marijuana?	**0.573**	**0.401**	**0.435**	−0.085	−0.079	0.166*	0.120	0.017	3
SP3. How many psychoactive drugs I use?	0.573	0.477	0.453	−0.066	−0.128	0.250*	0.107	0.085	0
SP4. How many prescribed drugs I use?^+^	**0.783**	**0.378**	**0.441**	−0.124	−0.213*	0.327*	0.201*	0.277*	**7^+^**
ANX1. Feeling nervous, anxious or on edge.	**0.881**	**0.777**	–	−0.352*	−0.474*	0.558*	0.438*	0.244*	3
ANX2. Not being able to stop or control worrying.^+^	**0.881**	**0.777**	–	−0.375*	−0.497*	0.563*	0.379*	0.220*	**4^+^**
DEP1. Feeling down, depressed, or hopeless.^+^	**0.897**	**0.806**	–	−0.474*	−0.558*	0.603*	0.531*	0.265*	**5^+^**
DEP2. Little interest or pleasure in doing things.	**0.897**	**0.806**	–	−0.443*	−0.499*	0.528*	0.544*	0.226*	
**Self-stigma of help seeking (59)**									
ST1. I would feel inadequate if I went to a therapist for psychological help.	**0.817**	**0.762**	**0.884**	−0.122	−0.013	0.045	0.057	−0.023	3
ST2. My self-confidence would NOT be threatened if I sought professional help.	0.691	0.661	0.891	−0.104	−0.048	0.102	0.101	0.033	0
ST3. Seeking psychological help would make me feel less intelligent.^+^	**0.803**	**0.746**	**0.886**	−0.142*	−0.035	0.111	0.097	−0.074	**4^+^**
ST4. My self-esteem would increase if I talked to a therapist.	0.393	0.376	0.907	0.001	−0.035	−0.099	−0.017	−0.037	0
ST5. My view of myself would not change just because I made the choice to see a therapist.	0.511	0.495	0.902	−0.136	−0.270*	0.197*	0.138*	0.044	3
ST6. It would make me feel inferior to ask a therapist for help.^+^	**0.901**	**0.838**	**0.879**	−0.157*	−0.056	0.080	0.169*	−0.037	**5^+^**
ST7. I would feel okay about myself if I made the choice to seek professional help	0.795	0.763	0.885	−0.116	−0.099	0.097	0.123	−0.018	0
ST8. If I went to a therapist, I would be less satisfied with myself.^+^	**0.826**	**0.785**	**0.884**	−0.083	−0.093	0.049	0.156*	−0.015	**4^+^**
ST9. My self-confidence would remain the same if I sought professional help for a problem I could not solve.	0.595	0.571	0.896	−0.235*	−0.224	0.217*	0.172*	−0.012	2
ST10. I would feel worse about myself if I could not solve my own problems.	0.593	0.560	0.898	−0.109	−0.100	0.108	0.200*	−0.005	1

^+^Indicates selected item based on highest number of criteria met. ^ + +^Indicates selected higher factor loading item if two items have same number of criteria met. FL, factor loadings; ITC, item-total correlation; AID, Alpha if item deleted; SE, self-efficacy; LS, life satisfaction; ST, stress; LNL, loneliness; DIS, disability; PE, positive emotion; EN, engagement; REL, relationships; ME, meaning; ACC, accomplishment; RES, resilience; OPT3, optimism; AC, academic problems; IP, interpersonal relationships problems; PP, physical health problems; SP, substance use problems; ANX, psychological health problems; ST, self-stigma of seeking help. Bold items are the ones that have good internal and external item qualities.

For the validation purpose, we used other tools as well. GPA was measured using the following single question “What is your current Grade Point Average?” The *Self-Efficacy for Academic Milestone Scale*; (60) was used to measure self-efficacy. *Perceived Stress Scale-4* (PSS-4) (61) was used to measure perceived stress. The *PROMIS^§^ Scale v1.2–Global Health Mental 2a* (62) was used to measure mental health quality of life. The *Oslo Social Support Scale* (63) was used to assess social support.

### Study 2

The Study 2 data, a part of funded large study, was collected from a Hispanic Serving Institution upon IRB approval. The principal investigator (PI) reached out to the Disability Center to collect data. The survey link via Qualtrics was shared electronically by disability office where students have been seeking counseling and accommodation services. All participants completed an online consent form before they started the survey. Participants received a $10 gift card upon completion of the study. We recruited 129 student veterans with disabilities (*M*_*age*_ = 33.05, SD = 8.80). The majority of participants were male (72.9%), white (81.4%), Hispanic (51.9%), and married (50.4%). About 97% of respondents reported they have a service-connected disability rating.

#### Measures

Sociodemographic variables were measured by demographic questionnaire. Grit was measured with the eight-item *Grit-S* (64). The Cronbach’s alpha coefficient for the Grit-S ranged from 0.83 to 0.84 (64). In the current study, the internal consistency reliability coefficient was found to be 0.69. Mental health QOL was assessed using the *PROMIS^^®^^ Scale v1.2–Global Health Mental 2a* (62). The Cronbach’s alpha coefficient in the present study was computed to be 0.75. Functional limitations were measured using the *World Health Organization Disability Assessment Schedule II* 12-item version (65). In the current study, the internal consistency reliability coefficient was found to be 0.91. PTSD symptoms were measured using the *PTSD Checklist for the DSM-5* (PCL-5) (66). The Cronbach’s alpha coefficient of the PCL-5 in the present study was computed to be 0.95. Dropout decisions were measured using the five-item *Intentions to Terminate University Studies or Switch Majors* (67). The Cronbach’s alpha coefficient of the scale in the present study was computed to be 0.82. COVID-19 stress was measured using the eight-item *Perceived Stress Questionnaire-8* (68), a shorter version of the *Perceived Stress Questionnaire-20* (69). The Cronbach’s alpha coefficient of the PSQ-8 in the present study was computed to be 0.83.

### Study 3

After receiving IRB approval for the Study 3, a part of funded large study, we contacted the student veteran office to collect data from participants. The student veteran office shared an online survey link with potential student veteran participants through email and social media. All participants completed an online consent form prior to accessing the survey. The Participants received a $20 gift card upon completion of the survey. For the Study 3, a total of 232 student veterans with PTSD symptoms (*M*_*age*_ = 28.43, SD = 5.42) were recruited for this study. Majority of participants were male (84.5%), White (71.6%), followed by 14.7% Black or African American, 9.9% American Indian or Alaska Native, 1.5% Asian, and 3.9% other. About 26% of student veterans identified themselves as being of Hispanic, Latino, or Spanish origin. Besides, majority of participants had served in the Army (46.6%), were full-time students (60.8%), were working (51.3%), and were using the GI Bill (70.7%). All participants reported that they had experienced a traumatic event, with at least one PTSD symptom measured by the *Primary Care PTSD Screen* for the DSM-5 (PC-PTSD-5) (70).

#### Measures

Sociodemographic variables were measured by demographic questionnaire. Overall health (i.e., “Overall, how would you rate your–overall health?”) and mental health QOL (i.e., “Overall, how would you rate your–mental health?”) was each measured with a single item. Similarly, participants’ GPA was measured by using a single item (i.e., “What is your current Grade Point Average?”). Functional limitations were measured using the World Health Organization Disability Assessment Schedule 2.0 (WHODAS 2.0) 12-item version (65). The WHODAS 2.0 12-item version was previously used for a veterans sample and demonstrated a strong internal consistency (α = 0.91) in a previous study with a veteran sample (71). The WHODAS 2.0 12-item version scores also demonstrated a strong internal consistency (α = 0.83) in the present study.

### Study 4

The final Study, Study 4, a part of funded large study, was approved by the IRB. Following approval, the PI contacted the disability and accommodation support office at a university located in a Southwest state. The disability and accommodation support office shared the survey link with college students with disabilities through an email invitation. Participants received a $10 USA gift card upon completion of the survey. A total of 105 college students with disabilities were recruited; however, however, we only retrieved the Hispanic participants for this study (*n* = 89; *M*_*age*_ = 26.13, SD = 8.11). The majority of participants were female (70.8%) and White (76.4%).

#### Measures

*Sociodemographic variables* were measured by demographic questionnaire. *Grit* was measured with the eight-item Grit-S (64). In the current study, the internal consistency reliability coefficient was found to be 0.79. *Mental health QOL* was assessed using the PROMIS^®^ Scale v1.2–Global Health Mental 2a (62). The Cronbach’s alpha coefficient in the present study was computed to be 0.80. Functional limitations were measured using the World Health Organization Disability Assessment Schedule II 12-item version (65). In the current study, the internal consistency reliability coefficient was found to be 0.83. *PTSD symptoms* were measured using the PTSD Checklist for the DSM-5 (PCL-5) (66). The Cronbach’s alpha coefficient of the PCL-5 in the present study was computed to be 0.96. *Dropout decisions* were measured using the five-item Intentions to Terminate University Studies or Switch Majors (67). The Cronbach’s alpha coefficient of the scale in the present study was computed to be 0.75. *COVID-19 stress* was measured using the eight-item Perceived Stress Questionnaire-8 (68), a shorter version of the Perceived Stress Questionnaire-20 (69). The Cronbach’s alpha coefficient of the PSQ-8 in the present study was computed to be 0.87.

### Data analysis

The *College Adjustment Index* was created following Bandalos (72) steps in scale development. The goal of the Study 1 is to identify and create an item pool from previously developed and psychometrically sound measurements. We used following recommended steps to select the psychometrically most sound items from measures (73): (a) internal item qualities (e.g., factor loadings, item-total correlation) and (b) external item qualities (e.g., items’ correlation coefficient with measures of other constructs). Internal item qualities refer to “properties of items that are determined in reference to scale itself” (p.169) (73). External item qualities refer to the relation of an item with measures of other constructs (73).

To select psychometrically most sound items, we selected items from the *PERMA-Profiler* (55), the BRS (56), the LOT-R (57), the ICP (13), the *Patient Health Questionnaire for Depression and Anxiety* (58), the *Self-Stigma of Seeking Help Scale* (59) by evaluating (a) each item’s factor loading, (b) item-total correlation, (c) each item’s effect on the internal consistency reliability of each scale, and (d) correlations with college life constructs.

Regarding internal item qualities, we conducted (a) a series of exploratory factor analysis (EFA) using principal axis factoring to identify factor loadings and (b) a series of item and reliability analysis to identify item-total correlations and each item’s effect on the internal consistency reliability of each scale. Regarding external item qualities, we conducted (a) a series of correlation analysis to identify the relationships between our selected scales’ items and the external correlates e.g., self-efficacy [the *Self-Efficacy for Academic Milestone Scale*; (60)], life satisfaction [the *Satisfaction with Life Scale* (SWLS) (74)], stress [the *Perceived Stress Scale-4* (PSS-4) (61)], loneliness [the *Three-Item Loneliness Scale* (75)], and disability (i.e., “Do you have a service-connected disability rating?”).

With Study 2, after identifying item pool from the Study 1, we conducted a series EFA with varimax rotation to identify the factor structure of the index score. Later, we conducted a parallel analysis via “psych” package (76) to identify and verify the number of factors of the index score. fa.parallel plots the eigenvalues for a principal components and the factor solution (76). Similarly, fa.parallel does the same for random matrices of the same size as the original data matrix.

The Study 3 and 4 were used to confirm construct validity of the index. The model structure of our scale was analyzed with a series of confirmatory factor analysis (CFA) via the “Lavaan” and “semPlot” packages (77, 78) for RStudio. We used all studies (Study 1–4) to confirm Study 3 factor structure. The following fit indices were used to evaluate the model fit: χ^2^ (not significant), the comparative fit index (CFI) (>0.90), Tucker–Lewis index (TLI) (>0.90) the standardized root mean residual (SRMR) (<0.08), and the root mean square error of approximation (RMSEA) (<0.08) (79–81). Finally, all studies were used to calculate internal consistency reliability coefficients and convergent and divergent validity.

We used all four studies to calculate coefficient omegas (ω) and coefficient alphas (α) for the sub scores and the total score. Finally, we run a correlation analysis to calculate divergent and convergent validity of the scale. Before we conducted analyses, we created z scores for all items given items were on different Likert rating scales. All statistical procedures were run via R Studio (82, 83) and SPSS 28.0.

## Results

### Internal and external item qualities

Table 1 demonstrates the detailed findings from the Study 1. Items were sorted based on their factor loadings. Our first criterion was to select items with the highest factor loading, highest item-total correlation, and lowest effect on the internal consistency reliability. After identifying items based on first criterion, we examined each item’s relation with external correlates to further identify psychometrically most sound items. Based on both criteria, we selected (a) a single item from each positive emotion[#PE1, (FL = 0.896; ITC = 0.829; AID = 0.856)], engagement [#EN2, (FL = 0.693; ITC = 0.428; AID = 0.378)], relationships [#REL2, (FL = 0.939; ITC = 0.804; AID = 0.721)], meaning [#ME1, (FL = 0.875; ITC = 0.824; AID = 0.882)], and accomplishment [ACC2, (FL = 0.874; ITC = 0.727; AID = 0.665)], (b) two-items from resilience [#RES1, (FL = 0.748; ITC = 0.679; AID = 0.811) and #RES4, (FL = 0.755; ITC = 0.684; AID = 0.808)], optimism [#OPT3, (FL = 0.726; ITC = 0.669; AID = 0.832) and #OPT6, (FL = 0.748; ITC = 0.680; AID = 0.830)], academic problems [#AP2, (FL = 0.744; ITC = 0.645; AID = 0.698) and #AP4, (FL = 0.880; ITC = 0.726; AID = 0.649)], interpersonal relationship problems [#IP1, (FL = 0.709; ITC = 0.611; AID = 0.724) and #IP4, (FL = 0.719; ITC = 0.611; AID = 0.724)], physical health problems [#PP1, (FL = 0.839; ITC = 0.723; AID = 0.749); and #PP3, (FL = 0.727; ITC = 0.653; AID = 0.784)], substance use problems [#SP1, (FL = 0.310; ITC = 0.273; AID = 0.623) and #SP4, (FL = 0.783; ITC = 0.378; AID = 0.441)], and psychological health problems [#ANX1, (FL = 0.881; ITC = 0.777; AID = NA) and #DEP2, (FL = 0.897; ITC = 0.806; AID = NA)], and (c) three-items from SSOSH [#ST3, (FL = 0.803; ITC = 0.746; AID = 0.886); #ST6, (FL = 0.901; ITC = 0.838; AID = 0.879); and # ST8, (FL = 0.826; ITC = 0.785; AID = 0.884)].

Each selected item was found to have small to large relation with selected external correlates in theoretically oriented direction. Relationships item#2 and item#3 had same criteria score; however, we selected item#2 given it had more optimal internal item qualities (e.g., higher factor loadings). Similarly, optimism item#3 and item#6 had same criteria score; however, we selected item#6 given it had more optimal internal item qualities (e.g., higher factor loadings).

### Exploratory factor analysis and parallel analysis

The Study 2 was used to measure factor structure of the scale via a series of EFA. The Kaiser-Meyer-Olkin value was 0.81 indicating a good degree of common variance among the variables and exceeding the minimum recommended value of 0.60 (84). The Bartlett Test of Sphericity was significant [χ^2^ (231, *N* = 127) = 1133.16, *p* < 0.001], indicating that correlations in the data set are appropriate for factor analysis. The Kaiser-Guttman rule (eigenvalue greater than one) was first used to determine the number of factors to be retained, followed by Cattell’s scree (85) test. Although the Kaiser-Guttman rule indicated a potential six factors, Cattell’s scree test yielded a five-factor measurement. Next, we conducted second EFA with five-factor solution. Table 2 represents results of the factor analysis. As a rule of thumb, we removed items that has multiple factor loadings (>0.40), insufficient factor loading (<0.30), and negative factor loading, resulting removing a total of four-items.

**TABLE 2:** Exploratory factor analysis (EFA) results of the student veterans with disabilities.

Items	Factor 1	Factor 2	Factor 3	Factor 4	Factor 5
To what extent do you lead a purposeful and meaningful life?	**0.778**				
I tend to bounce back quickly after hard times	**0.646**				
To what extent do you feel excited and interested in things?	**0.643**				
In general, how often do you feel joyful?	**0.639**	−0.403			
To what extent do you feel loved?	**0.632**				
How often do you achieve the important goals you have set for yourself?	**0.602**				
I’m always optimistic about my future.	**0.597**				
Overall, I expect more good things to happen to me than bad.	**0.514**				
Feeling lonely or isolated?		**0.678**			
Not being able to stop or control worrying		**0.674**			
Feeling down, depressed or hopeless		**0.614**			
Feeling like I’m not doing as well at school as I should?		**0.597**			
Trouble sleeping?		**0.575**			
Physical health problems?		**0.557**			
Problems with romantic or sexual relationships?		**0.536**			
It is hard for me to snap back when something bad happens.			−0.517		
How many prescribed drugs I use?			**0.470**		
My use of alcohol?			**0.426**		
It would make me feel inferior to ask a therapist for help.				**0.822**	
If I went to a therapist, I would be less satisfied with myself.				**0.522**	
Seeking psychological help would make me feel less intelligent.				0.341	
Difficulty caring about or concentrating on studies?		0.514			0.566

Bold items represent potential items for next analysis.

**FIGURE 1:**
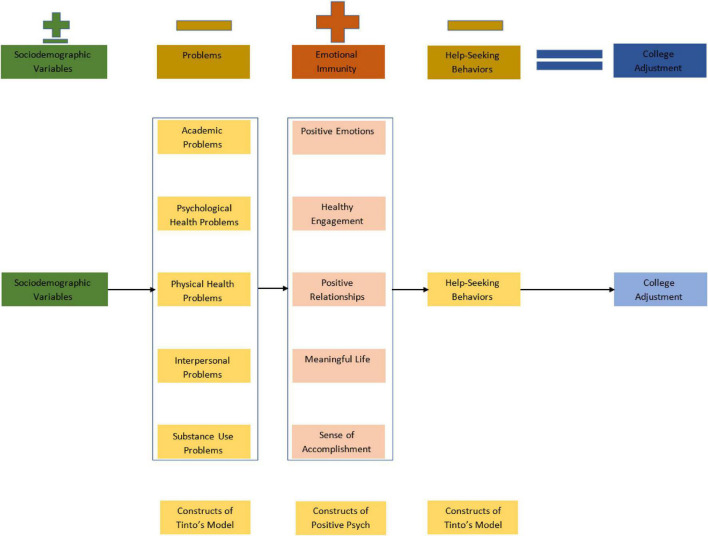
Parallel analysis results.

After removing identified items, we conducted a factor analysis, resulting in a four-factor structure; however, one factor had only one-item so that we run it again with three-factor structure. In order to confirm three-factor structure, we run a parallel analysis. The parallel analysis results yielded a three-factor structure (Please see Figure 1). Results revealed that the Kaiser-Meyer-Olkin value was 0.81 indicating a good degree of common variance among the variables and exceeding the minimum recommended value of 0.81 (84). The Bartlett Test of Sphericity was significant [χ^2^ (153, *N* = 127) = 815.36, *p* < 0.001], indicating that correlations in the data set are appropriate for factor analysis. The first factor was called as *emotional immunity* with a total of seven-items (e.g., “To what extent do you feel loved?”). The second factor was called as *common challenges* with a total of nine-items (e.g., “Feeling like I’m not doing as well at school as I should?”). Finally, the last factor was called as *help-seeking attitude* with a total of two-items (e.g., “It would make me feel inferior to ask a therapist for help.”). See Table 3 for detailed factor loadings.

**TABLE 3:** Modified EFA results of the student veterans with disabilities.

Items	Factor 1	Factor 2	Factor 3
To what extent do you lead a purposeful and meaningful life?	0.810		
To what extent do you feel excited and interested in things?	0.710		
To what extent do you feel loved?	0.660		
I’m always optimistic about my future.	0.609		
How often do you achieve the important goals you have set for yourself?	0.599		
Overall, I expect more good things to happen to me than bad.	0.564		
I tend to bounce back quickly after hard times	0.533		
Feeling down, depressed or hopeless		0.662	
Not being able to stop or control worrying		0.633	
Problems with romantic or sexual relationships?		0.615	
Feeling lonely or isolated?		0.606	
Feeling like I’m not doing as well at school as I should?		0.582	
Physical health problems?		0.561	
Trouble sleeping?		0.521	
How many prescribed drugs I use?		0.448	
My use of alcohol?		0.375	
If I went to a therapist, I would be less satisfied with myself.			0.627
It would make me feel inferior to ask a therapist for help.			0.580

### Confirmatory factor analysis

Exploratory factor analysis results were confirmed by using a series of confirmatory factor analyses (CFA). First, we used Study 3 results to independently cross-validate our Study 2 EFA results. The three-factor model generated a poor to acceptable fit: χ^2^ = 214.03, *df* = 132, χ^2^/*df* = 1.62, *p* < 0.05, CFI = 0.85, TLI = 0.82, SRMR = 0.07, and RMSEA = 0.05 (90% CI [0.06, 0.09]). Based on modification indices, conceptually and empirically meaningful correlated error terms were added to the model (86). The modified three-factor model generated a better fit: χ^2^ = 179.61, *df* = 129, χ^2^/*df* = 1.39, *p* < 0.05, CFI = 0.91, TLI = 0.89, SRMR = 0.06, and RMSEA = 0.04 (90% CI [0.02, 0.05]). The chi-square difference test indicated that the modified three-factor model fits the data significantly better than the non-modified three-factor model (Δχ^2^(3) = 34.43, *p* < 0.001).

We conducted three more CFAs using the Study 1 (SVDs), Study 2 (student veterans with disabilities), and Study 4 (Hispanic students with disabilities). The three-factor model generated a poor fit for the Study 1 that has a sample of SVDs: χ^2^ = 348.97, *df* = 132, χ^2^/*df* = 2.64, *p* < 0.05, CFI = 0.89, TLI = 0.88, SRMR = 0.06, and RMSEA = 0.08 (90% CI [0.07, 0.09]). Based on modification indices, conceptually and empirically meaningful correlated error terms were added to the model (86). The modified three-factor model generated a better fit: χ^2^ = 289.27, *df* = 130, χ^2^/*df* = 2.22, *p* < 0.05, CFI = 0.92, TLI = 0.91, SRMR = 0.05 and RMSEA = 0.07 (90% CI [0.06, 0.08]). The chi-square difference test indicated that the modified three-factor model fits the data significantly better than the non-modified three-factor model (Δχ^2^(2) = 59.69, *p* < 0.001).

Similarly, the three-factor model generated a relatively acceptable fit for the Study 2 that has a sample of student veterans with disabilities: χ^2^ = 228.59, *df* = 132, χ^2^/*df* = 1.73, *p* < 0.05, CFI = 0.88, TLI = 0.86, SRMR = 0.07, and RMSEA = 0.07 (90% CI [0.05, 0.09]). Based on modification indices, conceptually and empirically meaningful correlated error terms were added to the model (86). The modified three-factor model generated a better fit: χ^2^ = 195.68, *df* = 130, χ^2^/*df* = 1.50, *p* < 0.05, CFI = 0.92, TLI = 0.90, SRMR = 0.06 and RMSEA = 0.06 (90% CI [0.04, 0.08]). The chi-square difference test indicated that the modified three-factor model fits the data significantly better than the non-modified three-factor model (Δχ^2^(2) = 32.91, *p* < 0.001).

Finally, we tested this model with a different student body (Hispanic students with disabilities). The three-factor model generated a relatively acceptable fit for the Study 4 that has a sample of student veterans with disabilities: χ^2^ = 197.17, *df* = 132, χ^2^/*df* = 1.49, *p* < 0.05, CFI = 0.88, TLI = 0.86, SRMR = 0.08, and RMSEA = 0.07 (90% CI [0.05, 0.09]). Based on modification indices, conceptually and empirically meaningful correlated error terms were added to the model (86). The modified three-factor model generated a better fit: χ^2^ = 182.04, *df* = 130, χ^2^/*df* = 1.40, *p* < 0.05, CFI = 0.91, TLI = 0.89, SRMR = 0.07 and RMSEA = 0.06 (90% CI [0.04, 0.08]). The chi-square difference test indicated that the modified three-factor model fits the data significantly better than the non-modified three-factor model (Δχ^2^(2) = 15.13, *p* < 0.001). Table 4 represents the CFA findings.

**TABLE 4:** Confirmatory factor analysis (CFA) findings.

Model	χ^2^	Df	*p*	CFI	TLI	SRMR	RMSEA (90% CI)
Three-factor model (Study 1–Student veterans w/wo disabilities)	348.97	132	<0.05	0.89	0.88	0.07	0.09 (0.08, 0.10)
Re-specified three-factor (Study 1–Student veterans w/wo disabilities)	289.27	130	<0.05	0.92	0.91	0.05	0.07 (0.06, 0.08)
Three-factor model (Study 2–Student veterans with disabilities)	228.59	132	<0.05	0.88	0.86	0.07	0.07 (0.05, 0.09)
Re-specified three-factor (Study 2–Student veterans with disabilities)	195.68	130	<0.05	0.92	0.90	0.06	0.06 (0.04, 0.08)
Three-factor model (Study 3–Student veterans with PTSD symptoms)	214.03	132	<0.05	0.85	0.82	0.07	0.05 (0.04, 0.06)
Re-specified three-factor (Study 3–Student veterans with PTSD symptoms)	179.61	129	<0.05	0.91	0.89	0.06	0.04 (0.02, 0.05)
Three-factor model (Study 4–Hispanic students with disabilities)	197.17	132	<0.05	0.88	0.86	0.08	0.07 (0.05, 0.09)
Re-specified three-factor (Study 4–Hispanic students with disabilities)	182.04	130	<0.05	0.91	0.89	0.07	0.06 (0.04, 0.09)

Df, degree of freedom; CFI, comparative fit index, TLI, Tucker–Lewis index; SRMR, standardized root mean residual, RMSEA, the root mean square error of approximation; w/wo, with and without.

### Reliability

We used all four studies to calculate coefficient omegas (ω) and coefficient alphas (α) for the subscales. The first subscale called *emotional immunity* had coefficient alphas of 0.89 (Study 1), 0.86 (Study 2), 0.73 (Study 3), and 0.87 (Study 4). The first subscale called *emotional immunity* had coefficient omegas (ω) and coefficient alphas of 0.92 and 0.89 for the Study 1, 0.86 and 0.86 for the Study 2, 0.71 and 0.73 for Study 3, and 0.89 and 0.87 for the Study 4, respectively. The second subscale called *common challenges* had coefficient omegas (ω) and coefficient alphas of 0.84 and 0.84 for the Study 1, 0.81 and 0.81 for the Study 2, 0.68 and 0.69 for Study 3, and 0.75 and 0.76 for the Study 4, respectively. The third subscale called *help-seeking attitudes* had coefficient omegas (ω) and coefficient alphas of 0.85 and 0.86 for the Study 1, 0.60 and 0.61 for the Study 2, 0.39 and 0.40 for Study 3, and 0.48 and 0.44 for the Study 4, respectively. Table 5 represents reliability findings.

**TABLE 5:** Reliability findings.

Model	Study 1 F1	Study 1 F2	Study 1 F3	Study 2 F1	Study 2 F2	Study 2 F3	Study 3 F1	Study 3 F2	Study 3 F3	Study 4 F1	Study 4 F2	Study 4 F3	Study 1 total	Study 2 total	Study 3 total	Study 4 total
ω	0.92	0.84	0.86	0.86	0.81	0.61	0.71	0.68	0.39	0.89	0.76	0.48	0.90	0.82	0.60	0.85
α	0.89	0.84	0.85	0.86	0.81	0.60	0.73	0.69	0.40	0.87	0.75	0.44	0.90	0.84	0.66	0.85

α, Cronbach’s alpha; ω, omega reliability.

### Convergent and divergent validity

We conducted a series of correlation analysis to calculate convergent and divergent validity. Regarding the Study 1, emotional immunity factor was related to GPA (*r* = 0.21, *p* < 0.05), self-efficacy (*r* = 0.58, *p* < 0.05), social support (*r* = 0.53, *p* < 0.05), mental health QOL (*r* = 0.78, *p* < 0.05), and stress (*r* = −0.58, *p* < 0.05); common challenges factor was related to GPA (*r* = −0.21, *p* < 0.05), self-efficacy (*r* = −0.46, *p* < 0.05), social support (*r* = −0.42, *p* < 0.05), mental health QOL (*r* = −0.74, *p* < 0.05), and stress (*r* = 0.67, *p* < 0.05); and help-seeking attitudes factor was associated with social support (*r* = −0.21, *p* < 0.05). Regarding the Study 2, emotional immunity factor was associated with grit (*r* = 0.33, *p* < 0.05), mental health QOL (*r* = 0.57, *p* < 0.05), dropout decisions (*r* = −0.44, *p* < 0.05), PTSD (*r* = −0.46, *p* < 0.05), functional limitations (*r* = −0.32, *p* < 0.05), and COVID-19 stress (*r* = −0.48, *p* < 0.05); common challenges factor was associated with grit (*r* = −0.33, *p* < 0.05), mental health QOL (*r* = −0.26, *p* < 0.05), dropout decisions (*r* = 0.52, *p* < 0.05), PTSD (*r* = 0.72, *p* < 0.05), functional limitations (*r* = 0.61, *p* < 0.05), and COVID-19 stress (*r* = 0.59, *p* < 0.05); and help-seeking attitudes was associated with dropout decisions (*r* = 0.21, *p* < 0.05). In the Study 3, emotional immunity factor was correlated with GPA (*r* = 0.17, *p* < 0.05) and functional limitations (*r* = −0.18, *p* < 0.05) and common challenges factor was correlated with functional limitations (*r* = 0.35, *p* < 0.05). Finally, in the Study 4, emotional immunity was associated with grit (*r* = 0.46, *p* < 0.05), mental health QOL (*r* = 0.63, *p* < 0.05), dropout decisions (*r* = −0.42, *p* < 0.05), PTSD (*r* = −0.60, *p* < 0.05), functional limitations (*r* = −0.15, *p* < 0.05), and COVID-19 stress (*r* = −0.65, *p* < 0.05); common challenges factor was associated with grit (*r* = −0.36, *p* < 0.05), mental health QOL (*r* = −0.58, *p* < 0.05), dropout decisions (*r* = 0.33, *p* < 0.05), PTSD (*r* = 0.73, *p* < 0.05), functional limitations (*r* = 0.40, *p* < 0.05), and COVID-19 stress (*r* = 0.58, *p* < 0.05); and help-seeking attitudes was associated with dropout decisions (*r* = 0.27, *p* < 0.05) (Please see Table 6).

**TABLE 6:** Correlation coefficients among variables.

Constructs	Study 1 (*n* = 205)			Study 2 (*n* = 232)			Study 3 (*n* = 127)			Study 4 (*n* = 89)		
	Factor 1	Factor 2	Factor 3	Factor 1	Factor 2	Factor 3	Factor 1	Factor 2	Factor 3	Factor 1	Factor 2	Factor 3
**Study 1**												
GPA	0.214*	−0.209*	−0.002									
Self-efficacy	0.577*	−0.460*	−0.128									
Social support	0.533*	−0.421*	−0.208*									
Mental health QOL	0.780*	−0.735*	−0.107									
Stress	−0.575*	0.668*	0.069									
**Study 2**												
Grit				0.334*	−0.329*	−0.144						
Mental health QOL				0.573*	−0.256*	−0.137						
Dropout decisions				−0.439*	0.523*	0.214*						
PTSD				−0.456*	0.724*	0.031						
Functional limitations				−0.317*	0.613*	0.039						
COVID-19 stress				−0.476*	0.590*	−0.095						
**Study 3**												
GPA							0.166*	0.116	0.062			
Functional limitations							−0.179*	0.349*	0.122			
**Study 4**												
Grit										0.462*	−0.361*	−0.063
Mental health QOL										0.627*	−0.582*	−0.082
Dropout decisions										−0.416*	0.329*	0.266*
PTSD										−0.601*	0.730*	0.154
Functional limitations										−0.145	0.400*	0.075
COVID-19 stress										−0.645*	0.581*	0.097

* < 0.005.

## Discussion

**FIGURE 2:**
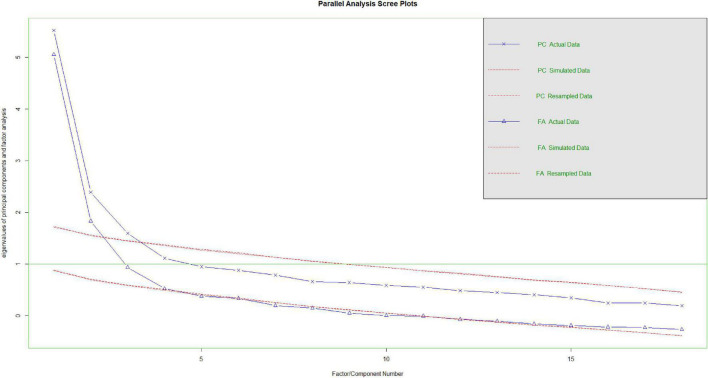
Extended student retention model called “Integrative Positive College Adjustment Model”. ± Represents both positive and negative impact on college adjustment; + represents positive impact on college adjustment; – represents negative impact on college adjustment.

This study utilized both constructs from Tinto’s model and positive psychology (see Figure 2 representing our new college adjustment model called “Integrative Positive College Adjustment Model”) to develop a new college adjustment index score and model for students with disabilities specifically for SVDs, but it could be also used for any college student with further model evaluation. College adjustment is significant challenge among SVDs due to transition-, disability, and psychosocial-related stressors. We believe this scale will meaningfully contribute to the fields of education and psychology by improving college adjustment among SVDs. Given student retention is impacted by multiple psychosocial and cognitive factors (87), our new, brief, and psychometrically sound scale may help professionals and researchers working with students including SVDs.

Our multisite and multiphase study used evidence-based approaches (72, 73, 88) to develop this index score to help clinicians and researchers working with college students including SVDs. Initially, we identified certain important domains (i.e., college and academic life problems, emotional health, and help-seeking behaviors) based on our previous work. After identifying these domains, with our Study 1, we used Stanton et al. (73)’s rigorous methods to shorten scales measuring the domains we identified. These scales included: the *PERMA-Profiler* (55), the BRS (56), the LOT-R (57), the ICP (13), the *Patient Health Questionnaire for Depression and Anxiety* (58), the *Self-Stigma of Seeking Help Scale* (59). Given these scales have been widely used in research and clinical practice, it can be concluded that our index score has an acceptable content validity. After carefully analyzing a total of more than 50-items, we selected 22-items to further analyze with a different sample of SVDs.

With Study 2, our results revealed that some items had poor item qualities. As a rule of thumb, we removed items with cross-loadings (i.e., 0.40 or more factor loading on two or more factors) (89). Eventually, our new EFA results yielded a three-factor solution, and we named these factors as *common challenges*, *emotional immunity*, and *help-seeking attitudes*. Next, we cross-validated our EFA findings using Study 1 to Study 3. CFA results revealed that modified three-factor model fit data well for SVDs. We also checked whether our index also have similar model fit in culturally different college student sample (Study 4) and found that our three-factor structure also fits data well for Hispanic college students with disabilities. Overall, although we tested construct validity only via factor analysis, our EFA and CFA findings supported construct validity of the index score.

We also tested reliability of the three factors generated from EFA and CFA. We calculated both Cronbach’s alphas and omega reliability scores. Our findings revealed that *common challenges* and *emotional immunity* factors had very good to excellent reliability scores for all four study samples. *Help-seeking attitudes* factor had relatively lower reliability scores compared to *common challenges* and *emotional immunity* factors. This could be partially due to lesser number of items given the number of test items affect the alpha values (90). However, our total index score was found to have acceptable to excellent reliability coefficients (0.60–0.90), indicating that the index has a good reliability.

Finally, we tested whether the index score is associated with certain similar and distinct constructs to examine convergent and divergent validity. Study 1 results revealed (a) higher levels of emotional immunity was associated with higher levels of GPA, self-efficacy, social support, mental health QOL, and lower levels of stress, (b) higher levels of common challenges was associated with lower levels of GPA, self-efficacy, social support, mental health QOL, and higher levels of stress, (c) higher levels of help-seeking attitudes factor was associated with lower levels of social support. Study 2 results revealed that (a) higher levels of emotional immunity was associated with higher levels of grit, mental health QOL, and lower levels of dropout decisions, PTSD, functional limitations, and COVID-19 stress, (b) higher levels of common challenges was associated with lower levels of grit, mental health QOL, and higher levels of dropout decisions, PTSD, functional limitations, and COVID-19 stress, and (c) higher levels of help-seeking attitudes factor was associated with higher levels of dropout decisions. Results of Study 3 demonstrated that higher levels of emotional immunity were associated with higher levels of GPA and lower levels of functional limitations. Results also revealed that higher levels of common challenges were associated with higher levels of functional limitations. Finally, Study 4 results revealed that (a) higher levels of emotional immunity was associated with higher levels of grit, mental health QOL, and lower levels of dropout decisions, PTSD, functional limitations, and COVID-19 stress, (b) higher levels of common challenges was associated with lower levels of grit, mental health QOL, and higher levels of dropout decisions, PTSD, functional limitations, and COVID-19 stress, and (c) higher levels of help-seeking attitudes was associated with higher levels of dropout decisions. These findings uniquely contribute to the literature by showing that our college adjustment index score was successfully associated with psychosocial, academic, and disability related outcomes.

Overall, this study aimed to create a college adjustment index score for students, including SVDs. This 18-item tool may help clinicians and researchers conceptualize college adjustment among students through the lens of integrative Tinto’s model and positive psychology approaches. Our psychometric analyses revealed that this index tool is brief, reliable, and valid tool to capture college adjustment in SVDs. Our scale will also help researchers and clinicians have a balanced practice given our scale measures emotional immunity, college problems, and help seeking attitudes, which is a holistic approach. This will also help researchers and clinicians have strength-based research and practice focusing on positive psychology factors such as grit, character strengths, gratitude, resilience in rehabilitation practice including psychiatric rehabilitation [e.g., (71, 91–95)].

Although our study is unique and has many strengths, this study has certain limitations. First, this study data includes only student veterans and Hispanic college students. Although majority of Veterans are White in our sample, we did not have an opportunity to collect a data from a more diverse student body. Therefore, our findings should be interpreted carefully by considering our samples. Second, these four studies were not planned to create this index score. Each study was funded by different agencies for different scope of work. However, this project has been a developing idea since dissertation focused on student veterans and college adjustment. Although rigorous and objective methods were applied to create this index score, a well-planned new study design would significantly benefit our current findings. Third, some of our samples consist of small sample size. This is partially due to difficulty reaching out this student body. Fourth, we created college adjustment index score or tool by selecting items via rigorous psychometric methods. We did not create our own item pool based on a focus group. Future work may also incorporate focus group and Delphi studies to create an item pool. Finally, some of our factors have low reliability scores which could be partially due to small numbers of items in these factors. For example, our help seeking subscale has only two-items, and it is expected that scales with less items have lower levels of reliability scores.

## Data availability statement

The datasets presented in this article are not readily available because due to IRB, data is not available. Requests to access the datasets should be directed to EU, the corresponding author.

## Ethics statement

The studies involving human participants were reviewed and approved by UT-El Paso and UW-Madison IRBs. The patients/participants provided their written informed consent to participate in this study.

## Author contributions

The author confirms being the sole contributor of this work and has approved it for publication.
